# The Level and Frequency of Faculty Incivility as Perceived by Nursing Students of Lorestan University of Medical Sciences

**DOI:** 10.25122/jml-2018-0055

**Published:** 2018

**Authors:** Maryam Mohammadipour, Shirin Hasanvand, Fateme Goudarzi, Farzad Ebrahimzadeh, Yadollah Pournia

**Affiliations:** 1. Student Research Committee, Lorestan University of Medical Sciences, Khorramabad, Iran; 2. Department of Nursing, Social Determinants of Health Research Center, Lorestan University of Medical Sciences, Khorramabad, Iran; 3. Department of Biostatistics and Epidemiology, Social Determinants of Health Research Center, Lorestan University of Medical Sciences, Khorramabad, Iran; 4. Faculty of Medicine, Lorestan University of Medical Sciences, Khorramabad, Iran

**Keywords:** Incivility, Nursing students, Faculty

## Abstract

**Background:** Little evidence exists concerning students’ perception of faculty incivility. Given the growing trend of incivility and the stressful nature of these behaviors, the present study aimed to investigate the level and frequency of faculty incivility as perceived by nursing students.

**Methods:** A stratified random sample of 178 students from four nursing faculties of Lorestan University of Medical Sciences participated in the present cross-sectional study. The Incivility in Nursing Education-Revised Survey, consisting of 24 items, was used to investigate the prevalence and frequency of faculty incivility, and the mean and frequency of each item was calculated separately. The data were analyzed using descriptive and analytical statistics (chi-square, independent t-test and analysis of variance), and the significance level was set at 0.05.

**Results:** Most of the participants were single, did not live in a dormitory, and ranged in age from 19 to 23. From their perspective, disrespect, physical threat, property damage, and physical violence were of paramount importance. 61.8% of them argued that they had “sometimes” and “always” experienced “unfair assessment” during the last year. Creating codes of behavior, enhancing awareness of civility, and developing and implementing policies for managing incivility were proposed as the most important strategies for improving civility.

**Conclusion:** Faculty members should be prepared for establishing friendly and respectful relationships, effective teaching, and applying a reality-based assessment. Identifying different and prevalent kinds of faculty incivility and making faculty members aware of them paves the way for faculty members to rethink their performance.

## Introduction

Incivility, a multidimensional and growing behavior [1], is a hotly-debated issue in nursing education [2], and one of the serious challenges of classrooms or clinical environments. According to Clark, incivility refers to students’ or professors’ disruptive and rude behaviors violating mutual respect [3]. In other words, incivility can be construed by disruptive behaviors which might lead to psychological or physiological distress, if they are left unattended [4].

Incivility might be found in student-student, faculty-student, or faculty-faculty relations [5]. Incivility is mostly a reciprocal process so that both students and faculty might contribute to its occurrence. However, nursing students, as one of the groups in the healthcare environment, hold little power and, accordingly, are more likely to experience incivility [6]. Stress caused by educational programs and the faculty’s unreasonable expectations is among the stress-inducing factors that students encounter, and they often feel helpless in dealing with them. Accordingly, such stressful experiences lead to conflict between the students and the faculty [7]. While mutual respect is necessary for successful and effective teaching and teachers play a crucial role in creating a respectful learning environment [8], faculty incivility is inevitable.

Faculty incivility is defined as any behavior disrupting learning or maintaining a positive classroom environment [9]. Faculty-student incivility takes different forms such as presenting lectures at a fast pace, having little interaction with students, standing aloof from students, doing unannounced assessments or asking unanticipated examination questions, arriving late to the classroom or canceling the class without informing students in advance and so forth [3, 10]. For example, in the study conducted by Rafiee Vardanjani et al. (2016), unfair grading and not being prepared for sessions (with 40.7%) were construed as the most disturbing behaviors in nursing education [8].

A review of the related literature reveals that student incivility has often been investigated, while few studies have been dedicated to investigating faculty incivility, and it has not received the attention it deserves. Nursing faculty contends that the prevalence and severity of student incivility are increasing [11], so that in Rafiee Vardanjani et al.’s (2016) study, from the faculty’s perspective, students’ uncivil behaviors (60%) exceeded faculty incivility (40%). However, students sustained that both students and faculty members equally contributed to uncivil behaviors [8]. To put it otherwise, as faculty members complained about students’ uncivil behaviors, students also made similar complaints about faculty members [12].

Students’ response to uncivil behaviors of faculty is feeling traumatized, helpless, and suffering from physical and psychological harm which consequently lead to experiencing emotional stress. Students experiencing uncivil behaviors from faculty feel undervalued, helpless and weak in dealing with problems, and this negatively affects their self-esteem [14]. Furthermore, students experiencing incivility feel anxious, develop physical symptoms, and have poor performance.

According to Schaeffer, the primary goal of nursing education is educating emphatic nurses [15]. However, incivility, either with significant or minor effects on students, hampers their progress and their ability in becoming an emphatic nurse [15, 16]. In fact, today’s students are tomorrow’s colleagues. Thus, if incivility is not controlled, students transform into employees with uncivil behaviors. Finally, their incivility threatens patients’ safety and care and leads to unemployment which finally causes ineffective caring [13, 17]. Furthermore, if incivility persists and is transferred from the learning environment to the workplace, it creates a poisoned working environment and brings about different problems in clinical environments, such as increasing the risk of making medical errors, leading to patient neglect, decreasing the quality of patients’ care, disrupting the interactions between members of the healthcare team, reducing patients’ safety and satisfaction [14, 18], and even reducing the self-esteem and productivity of the nursing staff [4].

In this regard, in a qualitative research entitled “Nursing Students’ Perception of Teachers’ Uncivil Behaviors”, Masoumpoor et al. (2017) contended that the adverse effects of disruptive behaviors could be subsumed under three major categories, namely “disruptive behaviors affecting communication climate”, “disruptive behaviors affecting ethical climate”, and “disruptive behaviors affecting learning climate” [10].

According to the preceding remarks, it can be concluded that teachers are neither superior nor inferior to students; instead, they are working collaboratively towards a common goal which is gaining knowledge. Hence, fair processes and interactions can lead students towards improving academic performance [19]. If the developed strategies aim at reducing incivility, improving the teaching-learning environment, improving postbellum faculty-student relations, and promoting a culture of civility in nursing education, investigating incivility is of paramount importance [11, 20]. Moreover, incivility has been construed as the main reason for the attrition of nursing students and nursing faculty [15]. Thus, given the importance of civil behaviors in improving individual, team, and organizational performance, the importance of investigating strategies for improving civil behaviors among nursing students to prepare them to meet the future of their job [21], and given the necessity of conducting more studies to scrutinize students’ perception of faculty incivility [22], the present study aimed to determine the importance and prevalence of faculty incivility from nursing students’ perspective.

## Materials and Methods

A sample of 178 students from four nursing faculties of Lorestan University of Medical Sciences participated in the present cross-sectional study. Using the formula 
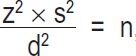
, the sample size was calculated as being 89 (with z=1.96, s=24, d=5), Then, by taking the design effect into account, the sample size was doubled (i.e., 178 individuals). The participants were selected using the stratified random sampling (each academic year was construed as a cluster with male and female students as its sub-clusters). In each sub-cluster, proportional to size, the participants were selected using the quota sampling until the required sample size was obtained. The criteria for selecting the participants were as follows: willingness to participate in the research, not being a visiting or guest student, and being among sophomore, junior, and senior students.

After providing explanations regarding the research, and obtaining informed and oral consent from the participants, the self-report survey of “the Incivility in Nursing Education-Revised (INE-R) Survey”, developed by Clark et al. (2014), was completed by the participants. The INE-R consists of three sections: the first section includes demographic items and the second section includes a list of 24 items of uncivil faculty behaviors and the respondents were asked to answer the survey using a 5-point Likert scale ranging from 1 to 4 (1: not uncivil, 2: somewhat uncivil, 3: moderately uncivil, and 4: highly uncivil). The respondents were also asked to indicate how often each behavior had occurred over the past 12 months using a 4-point Likert scale (1: never, 2: rarely, 3: sometimes, and 4: often). The mean and frequency were separately calculated for each item. Then, in four open-ended questions, the students determined the degree to which they identified incivility as a problem, and from 10 items, they selected the three top strategies for improving civility among nursing students [23]. The third section of the survey includes four qualitative fill-in-the-blank items in which the respondents were asked to provide a narrative description of their uncivil encounters and experiences, the chief reasons for incivility in nursing education, the most important consequences of incivility, and the most effective strategies for improving academic civility.

The above-mentioned scale is prevalently used in different studies, is translated into different languages and has high psychometric properties [23]. After corresponding with the developer and obtaining permission, the scale was translated into Persian through the backward and forward translation process and its validity and reliability were investigated. In the next stage, the scale was assessed using expert judgment (5 faculty members of nursing, 3 sociologists, and 3 psychologists). The content validity of the scale was estimated to be 0.9. To estimate reliability by the test-retest method, the scale was distributed among 30 nursing students and faculty members and was completed in 72 hours. The intra-class correlation coefficient was calculated to be 0.94. The internal consistency of this scale was evaluated using Cronbach’s alpha coefficient and was confirmed by the Cronbach’s alpha of 0.94.

To analyze the data, SPSS software, descriptive statistics (mean, frequency, and percentage frequency), and analytical statistics (chi-square, independent t-test, and analysis of variance (ANOVA)) were used and the significance level was set at 0.05. The third section of the scale was analyzed using the six-phase method proposed by the developer of the scale. The ethical approval was obtained from the Committee of the Research Vice-Chancellorship of Lorestan University of Medical Sciences (Project Number: lums.REC.1395.197).

## Results

A sample of 178 students from four nursing faculties of Lorestan University of Medical Sciences participated in the present cross-sectional study. Most of the participants were single (91%), did not live in a dormitory (56.2%), and ranged in age from 19 to 23 (81.6%). Additional demographic information is provided in Table 1.

The mean score of the students’ perception of faculty incivility is provided in Table 2. As the table indicates, items 21, 22, 23, and 24 had the highest means, which is indicative of the high level of incivility of the putative behaviors. In contrast, items 5, 8, 15, and 18 had the lowest means.

The frequency of uncivil faculty behaviors is depicted in Table 3. Most of the students contended that they had “sometimes” and “always” experienced “unfair grading”. 14.6% had experienced “insincerity and coldness towards others”, and 14% had always experienced “inefficient or ineffective teaching methods” and “refusing to talk about re-examinations, test deadlines, or revising grades”. In contrast, 69.7% and 44.9% of the participants had never experienced “sending inappropriate or impolite emails to others” and “mentioning discriminative ideas (racial, ethnic, gender and others)”, respectively.

**Table 1:** Students’ demographic information

Variables	Category	Frequency	Percentage frequency
Age	19-23	163	81.6
	>23	15	18.4
Gender	Male	96	53.9
	Female	82	46.1
Marital status	Single	162	91
	Married	16	9
Residence status	Dormitory	78	43.8
	Non-dormitory	100	56.2
University entering year	2013	29	16.3
	2014	45	25.3
	2015	66	37.1
	2016	38	21.3
Semester	3-4	76	42.7
	5-6	56	31.5
	7-8	46	25.8
Average of passed courses	<16	37	19.1
	16-17.99	107	60.1
	≥18	34	20.8
Department (faculty)	Khorramabad	81	45.5
	Aligoudarz	27	15.2
	Poldokhtar	36	20.2
	Boroujerd	34	19.1

**Table 2:** Mean score of the nursing students’ perception of faculty incivility

	Items	Mean ± SD
1	Expressing disinterest, tiredness, or indifference toward the course content or the subject matter	3.20±1.02
2	Impolite gestures or nonverbal behaviors towards others (e.g., eye rolling, pointing the finger at somebody and others)	3.5±0.77
3	Inefficient or ineffective teaching methods (changing the time of assignments or exams)	3.2±0.89
4	Refusing or being reluctant to answer direct questions	3.1±0.90
5	Using a computer, cell phone, or any other media in faculty meetings, councils, or doing irrelevant activities	2.1±0.89
6	Delay in entering the class or other planned activities	2.9±0.83
7	Early leaving of classes or other planned activities	2.7±0.86
8	Not being prepared for the class or other planned activities	2.1±0.92
9	Cancelling the class or other planned activities without prior announcement	3.3±0.96
10	Insincerity and coldness towards others (indifference and not accepting students’ opinions)	2.9±0.88
11	Punishing the whole class because of one student’s inappropriate behavior	3.7±0.78
12	Permitting students’ marginal talks which disturb the class	3.1±0.84
13	Unfair grading	3.8±0.59
14	Spoiling or being impolite to others	3.8±0.65
15	Refusing to talk about re-examinations, test deadlines, or revising grades	2.1±0.89
16	Ignoring, indifference to or encouraging disturbing student behaviors	3.1±0.84
17	Showing superiority, abusing position or personal rank (such as threatening students not to pass the exam without any reason)	3.5±0.77
18	Inaccessibility outside of the class (not answering contacts or emails or not attending the workplace during work hours)	2.1±0.84
19	Sending inappropriate or impolite emails to others	3.8±0.69
20	Mentioning discriminative ideas (racial, ethnic, gender and others)	3.6±0.75
21	Disrespecting (scorning or swearing) others	3.9±0.55
22	Threatening others physically (implied or real)	3.9±0.58
23	Damaging properties	3.9±0.58
24	Making threatening statements about weapons	3.9±0.59

With regard to the relationship between the demographic information and the mean score of the students’ perception of uncivil faculty behaviors, the findings demonstrated that there was a significant relationship between the mean score of the students’ perception of uncivil faculty behaviors and the average of the grades of their passed courses (0.002) so that the mean score of the students’ perception of uncivil faculty was higher among the students whose average was below 16, compared to the students whose average was above 16. In a nutshell, the students with lower averages reported a higher level of incivility in faculty behaviors. However, regarding other characteristics, there was no significant relationship.

There was a significant relationship between the frequency of the students’ perception of faculty incivility and the students’ gender (0.025) and residence status (0.009). However, for other characteristics, no significant relationship was observed. The prevalence or frequency of faculty incivility was reported to be higher among the female students living in dormitories compared to the male students not living in dormitories.

According to the results of the other sections of the scale adopted in the present study, from the students’ perspective, incivility was construed as a serious problem in nursing education. Comparing the incidence of faculty incivility and student incivility from the students’ perspective revealed that the possibility of the incidence of incivility was higher among the students. From the students’ perspective, the three important strategies for promoting civility in nursing education included determining codes of conduct defining acceptable and unacceptable behaviors (28.7%), raising awareness and providing civility-related education (29.8%), and developing and implementing comprehensive policies and procedures for managing uncivil behaviors (9.6%) (Table 4).

**Table 3:** Table 3: Frequency of faculty incivility from the students’ perspective

	Frequency (Percentage frequency)			
Items	Often	Rarely	Sometimes	Never
1	36(20.2)	82(46.1)	47(26.4)	13(7.3)
2	45(25.3)	80(44.9)	45(25.3)	8(4.5)
3	16(9)	67(37.6)	70(39.3)	25(%14)
4	30(16.1)	86(48.3)	55(30.9)	7(3.9)
5	40(22.5)	86(48.3)	40(22.5)	12(6.7)
6	22(12.4)	91(51.1)	56(31.5)	9(5.1)
7	29(16.3)	86(48.3)	54(30.3)	9(5.1)
8	32(18)	90(50.6)	49(27.5)	7(3.9)
9	27(15.2)	63(35.4)	68(38.2)	20(11.2)
10	14(7.9)	72(40.4)	66(37.1)	26(14.6)
11	32(18)	73(41)	55(30.9)	17(9.6)
12	36(20.2)	77(43.3)	55(30.9)	9(5.1)
13	19(10.7)	49(27.5)	70(39.3)	40(22.5)
14	56(31.5)	71(39.9)	41(23)	10(5.6)
15	17(9.6)	77(43.3)	59(33.1)	25(14)
16	34(19.1)	90(50.6)	42(23.6)	12(6.7)
17	40(22.5)	72(40.4)	51(28.7)	15(8.4)
18	23(12.9)	77(43.3)	56(31.5)	21(11.8)
19	24(69.7)	33(18.5)	13(7.23)	8(4.5)
20	80(44.9)	68(38.2)	20(11.2)	10(5.6)
21	144(80.9)	24(13.5)	9(5.1)	1(0.6)
22	172(96.6)	4(2.2)	1(0.6)	1(0.6)
23	173(97.2)	4(2.2)	1(0.6)	0
24	174(97.8)	2(1.1)	2 (1.1)	0

In the third section of the scale, the respondents were asked to narrate their uncivil encounters and experiences over the past 12 months, the significant reasons for incivility in nursing education, the most important consequences of incivility, and the most effective strategies for improving academic civility. The results of the analysis of the participants’ descriptions are provided in Table 5.

## Discussion

The present study aimed to investigate the importance and prevalence of faculty incivility as perceived by nursing students. The findings indicated that items 21 (disrespecting – scorning or swearing – others), 22 (threatening others physically – implied or real), 23 (property damage), and 24 (making threatening statements about weapons) had the highest means. This is suggestive of the importance of these behaviors from the students’ perspective, and this finding is in line with the findings of the Korean study carried out by De Gagne et al. (2016) demonstrating the paramount importance of “threats of physical harm against others” and “making threatening statements about weapons” [24]. As cogently put by Kanami (2017), the intensity of incivility is less than bullying and aggression [25]. However, the finding of the present research belies this conception of incivility in that, as it can be observed, in the present study, the students construed items with the theme of aggression as having high levels of incivility. In fact, in this study, “threats of physical harm” was conceived of as being threatening and was considered among uncivil behaviors although the students had not experienced such behaviors. This is corroborative of the argument that being threatening is not tantamount to displaying that behavior [26].

In contrast, items 5 (using a computer, cell phone, or any other media in faculty meetings, councils, or doing irrelevant activities), 8 (not being prepared for the class or other planned activities), 15 (refusing to talk about re-examinations, test deadlines, or revising grades) and 18 (inaccessibility outside of the class – not answering contacts or emails or not attending workplace during work hours -) had the lowest means. Although it is repeatedly borne out that being unprepared for class leads to inefficient teaching [27], in this study, from the students’ perspective, professors not being prepared for the class had a lower level of incivility, compared to other behaviors. However, this finding contradicts the results of a study carried out by Clark (2008) in which “being unprepared for class or other scheduled activities” was regarded as incivility. In a Chinese study, likewise, “being unprepared for class or other scheduled activities” was perceived as the most prevalent uncivil behavior (82.4%) [24]. It is evident that this finding contradicts the findings of the present study. The observation that this item has a low mean score in this study can be attributed to the lower incidence of this behavior from the students’ perspective in that 3.9% of the participants had experienced this behavior.

One of the uncivil behaviors most of the students had “always” or “often” experienced was “unfair grading”. This finding is in agreement with the findings of Delperato (2013) and Muliira et al. (2017) [28, 3]. In the findings of Muliira et al.’s (2017) study, besides other uncivil behaviors, unfair grading was among the most prevalent uncivil behaviors. In the phenomenological research performed by Delperato (2013), students’ personal experiences of faculty incivility revealed that incivility is described in terms of the four themes of “experiences of contempt, unfair grading, setting unrealistic expectations, and ignoring students”, and it goes without saying that it agrees with the present study’s findings. Furthermore, in Juibari et al.’s (2010) research, 67.1% of the students had sometimes experienced “unfair grading”. As the preceding remarks confirm, in previously-conducted studies, grading has always been a challenging issue for both students and teachers [24]. Teachers should always adopt a uniform grading policy [29]; otherwise, injustice leads to unfair competition among students [30].

**Table 4:** Frequency distribution of the three dominant strategies for promoting civility

Strategy content	Chosen as the first priority	Chosen as the second priority	Chosen as the third priority	Not selected	Standard deviation± mean of the specified priority
	Frequency (Percentage frequency)	Frequency (Percentage frequency)	Frequency (Percentage frequency)		Mean ± SD
Using experimental instruments (such as questionnaire to evaluate incivility and pay attention to progress/strong points	30(16.9)	0	0	148	3.49±(1.12)
Determining codes of behavior for distinguishing acceptable behaviors from unacceptable behaviors	51(28.7)	1(0.6)	0	126	3.12±(1.36)
Being a role model from civility and professional perspective	44(42.7)	15(8.4)	0	119	3.08±(1.32)
Increasing awareness regarding civility	38(21.3)	53(29.8)	4(2.2)	83	2.7±(1.24)
Taking civility into account in evaluating faculty	8(4.5)	25(14)	4(2.2)	141	3.56±(0.89)
Teaching effective relations and effective discussing of differences	6(3.4)	34(19.1)	16(9)	122	3.42±(0.91)
Implementing policies for managing incivility	0	26(14.6)	17(6.9)	135	3.61±(0.72)
Encouraging civility professionalism	1(6)	18(10.1)	30(9.16)	129	3.61±(0.68)
Implementing a strategy for reducing stress and for increasing self-care	0	6(3.4)	25(14)	147	3.79±(0.48)
Accepting personal responsibility and being responsible for one’s behaviors	0	0	82(1.46)	96	3.53±(0.49)

Furthermore, according to the World Health Organization, establishing effective communication with students is one of the major competencies of nursing instructors and, in some studies, fostering effective student communication is reported as a key strategy for reducing challenging behaviors. However, in this research, 14.6% of the respondents had always experienced professors’ “insincerity and coldness towards others”. In fact, the findings of most studies highlight the importance of professors’ communication skills. For example, in Mobasheri et al.’s (2011) study, establishing an intimate faculty-student relationship is introduced as one of the characteristics of a good teacher from the students’ perspective [32].

A considerable percentage of the participants had always experienced “ineffective or inefficient teaching method” and “refusing to discuss make-up exams, extensions, or grade changes”. The participants of Clark et al.’s (2008) study also had perceived ineffective teaching methods as one of the faculty’s uncivil behaviors. The importance of ineffective teaching methods is highlighted when considering the fact that it can affect students’ participation and consequently lead to incivility [22]. The argument that the professors’ teaching methods and students’ degree of participation are intertwined is substantiated in other studies. In Clark et al.’s qualitative research, the participants viewed ineffective teaching methods as the most prevalent uncivil behavior [33]. Besides, in Yassour-Borochowitz’s (2016) research, ineffective teaching methods were perceived as one of the most problematic uncivil faculty behaviors [34]. The findings also demonstrated that most of the participants sustained that they had never experienced “sending inappropriate or impolite emails to others” and “disrespecting (scorning or swearing) others”. This observation can be ascribed to the limited interaction and correspondence between students and faculty during the undergraduate program.

**Table 5:** Descriptive narration of the students’ incivility experiences

Example of incivility experiences	Main reason for incivility incidence	Consequences of incivility	Strategies for promoting civility
Faculty’s inappropriate behavior	Being unprepared	Aggression	Increasing faculty’s and students’ awareness of civility
Disdaining students	Exerting superiority	Lack of academic and practical knowledge	Teaching friendly and effective relations
Unfair grading	Not establishing effective faculty-student relationship	Disrespectful behaviors	Accepting personal responsibility
Leaving classroom without any reasons	Cultural differences	Not establishing effective faculty-student relationship	Using previous experiences
Misusing power	Faculty’s lack of experience	Educating inefficient workforce	Having reward and punishment systems
Being engaged in side conversations	Heavy workload	Distraction	Being a role model
Indifference	Gender gap	Harming self and others	Discussing differences and conflicts
Exhibiting racial behaviors	Lack of culture	Weakening their professional status	Generating motivation
Expressing superiority	Lack of management	Transferring incivility from academic to working environment	Promoting justice
Threatening to fail a student	Inappropriate behavior patterns	Lack of motivation	Reducing stress
Exhibiting disrespectful behaviors	Unhealthy environments	Feeling anxiety and stress	Determining acceptable codes of conduct
Using cell phone	Moral weakness	Forgetting the purpose of education	Developing comprehensive policies for managing incivility
Exhibiting inappropriate behaviors	Lack of motivation	Poisoning the working environment	Efficient teaching
Being inflexible	Not accepting criticism	Reducing the quality of nursing care	Being precise in teacher selection
Not being honest		Being engaged in side issues	Self-care
	Main reason for incivility incidence	Poisoning the working environment	Strategies for promoting civility

In this study, according to the students’ descriptions, it was revealed that incivility is a significant problem in nursing education. In the study carried out by Ibrahim et al. (2016), the intensity of student incivility was very high and was interpreted as one of the main causes of academic failure [35]. In Marchiondo et al.’s (2010) study, the prevalence of incivility was also reported as being 50% [36]. However, in few studies, for example by Natarajan et al. (2017), and Muliira et al. (2017) performed in Oman, the incidence of faculty incivility was low, and the student academic incivility was moderately present in nursing education [37, 3]. Although the incidence of incivility was low, given that it affects all aspects of academic environments, planning to manage them is of paramount importance and nursing faculty are primarily expected to be an example for each other, students, and for health care teams [38].

The results of investigating the relationship between the incidence of incivility and demographic information revealed that the incidence and frequency of faculty incivility were higher among the female students living in dormitories, compared to the male students not living in dormitories. Stork and Hartly (2009), in line with the present study, demonstrated that male students’ perception of faculty incivility is lower than that of female students [39].

The results obtained in the present study regarding effective strategies for promoting civility in nursing education are in line with the results of previously-conducted studies on this subject. According to Clark et al. (2012), increasing faculty and students’ awareness of incivility plays a crucial role in promoting civility in academic environments [33]. Rad et al. (2016) emphasized organizing and holding workshops to increase academic practitioners’ awareness of incivility [14].

The findings of the qualitative section of the research, presented in Table 4, agree with the findings of the quantitative section and existing evidence. One of the significant examples of an uncivil encounter was teachers misusing their power (or, “rankism”, using Clark’s (2008) terminology). In Clark (2007), all of the participants felt powerless and helpless and identified the faculty’s power as the leading cause of incivility. Accordingly, identifying rankism and developing some strategies for refuting it to improve student-teacher interaction is of paramount importance [26].

Regarding the factors for the incidence of incivility, gender gap, as it was highlighted in Ziefle’s (2018) study, was emphasized by participants [40]. In their narrative descriptions regarding the consequences of incivility, students referred to incivility consequences which were repeatedly mentioned in the literature on this issue, such as decreasing the quality of patients’ care, increasing the risk of patient injury, making medical errors, feeling anxious and stressed [13, 16], interfering with teaching and learning, transferring incivility from the academic environment to the working environment, and losing one’s job [17]. Thus, it can be argued that the findings of the present study are corroborative of the findings of previous studies. Furthermore, strategies for promoting civility in nursing education were in line with the strategies in the literature. However, justice, being precise in teacher selection, and improving nursing education culture, which are not mentioned in previous studies, are mentioned in the present study.

## Conclusion

The findings of the present study are corroborative of the critical role of faculty members in fostering civility culture in clinical and academic environments. Faculty members are required to be prepared for establishing an intimate, friendly and respectful relationship with their students, effective teaching, and fair grading. Moreover, identifying prevalent uncivil behaviors and increasing the faculty’s awareness of incivility paves the way for them to rethink their performance and create a constructive environment for teaching and learning.

## Acknowledgments

Our sincere appreciation goes to all nursing teachers and students of the Lorestan University of Medical Sciences who participated in the present study.

## Conflict of Interest

The authors confirm that there are no conflicts of interest.
